# Application of Collagen-Based Scaffolds for the Treatment of Spinal Cord Injuries in Animal Models: A Literature Update

**DOI:** 10.7759/cureus.25997

**Published:** 2022-06-16

**Authors:** Dimitrios Zachariou, Dimitrios Stergios Evangelopoulos, Meletis Rozis, Eftychios Papagrigorakis, Athanasios Galanis, Michail Vavourakis, Spyros G Pneumaticos, John Vlamis

**Affiliations:** 1 3rd Orthopaedic Department, National and Kapodestrian University of Athens, KAT General Hospital, Athens, GRC

**Keywords:** collagen-based scaffold, scaffold, tissue engineering & regenerative medicine, spinal cord regeneration, animal model, spinal cord injury (sci)

## Abstract

SCI is regarded as one of the most devastating central nervous system (CNS) injuries, exhibiting an alarmingly rising incidence rate, indirectly connected with the expansion of the global economy. The consequences of SCI are multidimensional: SCI injuries may result in permanent voluntary motor dysfunction and loss of sensation while incurring heavy economic and psychological burdens as part of the treatment. Thus, it is crucial to develop effective and suitable SCI treatment strategies. Collagen-based scaffold application is one of the most promising methods of SCI treatment.

This review compiles newer bibliographical data regarding the application of collagen scaffolds for the treatment of Spinal cord injury (SCI) in animal models. Recently, several relevant studies have been carried out using carefully selected animals with similar pathophysiology to humans. In mouse, rat and canine models that have undergone transection or hemisection, the stump connection, the transplanted cell differentiation, and the elimination of glial scar are promising. Also, encouraging results have been found regarding the increased neuronal growth, the decreased collagen deposition, the behavioral recovery, the improved electrophysiology, and the enhanced axonal regeneration.

## Introduction and background

Spinal cord injury (SCI) is regarded as one of the most devastating central nervous system (CNS) injuries, exhibiting an alarmingly rising incidence rate, indirectly associated with the expansion of the global economy [1-5]. The consequences of SCI are multidimensional: SCI injuries may result in permanent voluntary motor dysfunction and loss of sensation while incurring heavy economic and psychological burdens. Thus, developing effective and suitable SCI treatment strategies is crucial [1].

SCI causes neurological disabilities as the CNS central axons or the nerve fibres often fail to regenerate [4,6] due to chronic inflammatory response, demyelination, and increased proteoglycans [6]. In mammalian CNS, the leading cause behind the limitation of central axons to regenerate is glial scar formation, which inhibits axonal remodelling and regrowth [4,7]. Healthy glial cells support neuronal function and signal transmission [8]. However, when SCI occurs, the borders of SCI lesion are separated from healthy tissue by mechanical damage and secondarily by a glial scar densely populated by newly hypertrophic and proliferating cells [4]. These cells are mainly astrocytes, pericytes, non-pericyte perivascular cells, and Schwann cells [4]. This process hinders the regeneration of neurons’ myelin sheath and function, with glial scar constituting a physical and molecular barrier to developing CNS axons [8,9].

Most therapeutic strategies developed in recent years focus on eliminating post-SCI inhibitors of regeneration. Contemporary approaches provide support and guidance toward the regeneration of affected neurons by applying neural scaffolds to the SCI lesion site [1]. Advanced tissue engineering (TE) technology has paved the way for SCI treatment [3,10,11]. The extracellular matrices (ECMs) allow living cell inoculation, growth, and differentiation, thus promoting axon and fibre regeneration. Matrices are co-cultured with cells and transplanted in the SCI area [4]. This way, the ECM of the spinal cord can be successfully mimicked, as scaffolds are rich in glycosaminoglycan, a gap-filling polysaccharide of staunch structure [4]. However, excessive amounts thereof contribute to the uncontrollable development of extensive and grave glial scar. The most suitable solution to combat this issue shall be collagen [4].

There is a great deal of attention regarding the characteristics of scaffolds, especially the biomaterials used to construct them. Wang et al. stress the importance of biocompatibility for cells, apposite porosity topography and permeability of scaffold materials [12]. As aforementioned, collagen-based scaffolds are a popular choice for biomaterials used for SCI treatment purposes. Collagen is a protein found in abundance in the ECM, provoking a minimal autoimmune response and promoting cell adhesion, proliferation, migration, and differentiation [13]. Collagen scaffolds come in various forms, including hydrogel, sponge, or guidance conduit, which serves as an instrument to administer therapeutic drugs and proteins to the SCI site [14]. This review constitutes a compilation of newer bibliographical data on collagen scaffolds as applied for SCI treatment purposes in animal models to provide a fresh insight into the available bibliography.

## Review

Pathophysiology of SCI

The pathophysiology of SCI is often broadly categorized as either “acute impact” or “compression”. Injury resulting from acute impact is essentially a spinal cord concussion that triggers a series of reactions localized in the grey matter, concluding in haemorrhagic necrosis. Grey matter hypoperfusion usually triggers the sequence of events mentioned in this section. Reperfusion and increased intracellular calcium occur soon after the injury and are crucial for the injury outcome. Thus, mechanisms transpiring in the initial stages of injury should be targeted for improved prognosis. Injury resulting from spinal cord compression occurs upon impingement of the spinal cord by a mass, increasing the parenchymal pressure. As far as the white matter is involved, the tissue response is gliosis, demyelination and axonal loss. At the same time, grey matter structures are preserved. A rapid or critical degree of compression will collapse the venous side of the microvasculature, resulting in vasogenic oedema and exacerbated parenchymal pressure. Therefore, leading to swift progression of the SCI process.

Collagen scaffolds for SCI treatment

In line with the above, several regeneration medicines (RM) and TE studies prove the effectiveness of injected collagen hydrogels. Notably, Sugai et al. in 2015 performed a transection of the spinal cords in rats and compared the effect of collagen-based scaffolds with fibrin and Chitosan. The collagen-based scaffolds showed the most proliferation after the transplant, while the neural stem and progenitor cells survived up to 11 weeks after the transplant [15]. 

Breen et al. in 2016 examined the role of injectable collagen hydrogel in administering neurotrophin-3 into rat models undergoing a lateral hemisection of the spinal cord between T9-T10. According to Breen et al., functional recovery was significantly increased at four weeks postoperatively. The NG2 positive cells expressed within the lesion area were meaningfully reduced compared to the hemisection-only group. Additionally, they reported lowered macrophage and microglial response and reduced glial scarring of the SCI area [14]. 

Han et al. in 2017 studied the effect of linear ordered collagen scaffolds loaded with human mesenchymal placenta stem cells in canines with a complete spinal cord transection. Han et al. reported many host cells in the collagen scaffold group, while the new tissue was in a structured form. Contrary, the regenerated tissue was in structural disorder in the ungrafted group. Additionally, they reported an increase in the number of neurons and regenerated axonal fibers penetrating the lesion site with linear order and distinct distribution [16]. 

Li et al. have also presented a series of outstanding pieces of research on regeneration and overcoming inhibitory factors following SCI [17-20]. In 2015, they analyzed the delivery of proteins and drugs through scaffolds to enhance post-SCI recovery in rats, proving that collagen scaffolds could support the regeneration of the axons and their remyelination. They also showed that the rats that received the collaged-based scaffolds modified by CBD-EphA4LBD and CBD-PlexinB1LBD promoted the development of more axon fibres through the lesion site. The rats in that group exhibited significant improvements in locomotion from the first week [17]. 

In 2016, the same group of scientists presented a porous collagen scaffold with neurotrophic factors CBD-BDNF and CBD-NT3. They reported that the scaffold promoted the outgrowth of the cerebellar granular neurons in vitro. They also found that the cavities caused by the SCI injury were significantly reduced and suggested that the functionalized collagen scaffold could enhance the anti-inflammatory function. Combined with Cyclic Adenosine Monophosphate (cAMP), the scaffold aided the repair of a completely transected spinal cord in a rat model. Still, the locomotion outcome was unsatisfactory, suggesting that rebuilding an injured neural connection is exceptionally complex [18].

In 2017, Li et al. studied the effect of functional collagen scaffold with Cetuximab in rats and dogs with T8 SCI. The Modified Linear Order Scaffolds with Cetuximab showed a much higher number of newborn and mature neurons in rodents and dogs. Additionally, they reported that the cetuximab group had the highest distribution and density of neuronal nuclei, meaning that the functional scaffold promotes migration and neural production [19].

Most recently, Li et al. demonstrated that paclitaxel (PTX) reduced glial scarring attributed to SCI by rescuing myelin-inhibited differentiation of NSCs. The cells were co-cultured with PTX and transplanted via a functional collagen scaffold into a complete T8 transection of the spinal cord in a rat model. Improvement of sensation and locomotion was confirmed by Western Blot (WB) and mRNA-Seq results that showcased the ability of PTX to trigger neuronal differentiation via the Wnt/β-catenin signalling pathway [20]

The effect of collagen-based scaffolds to release therapeutic substances was also discussed earlier by Snider et al., whereby the effectiveness was demonstrated using rat models to provide relevant evidence during the acute and chronic SCI phase. As mentioned by Snider et al., the control group showed slight movement in one or two joints and extensive movement in one. Additionally, they reported reduced inflammatory cells and a higher organization in the new tissue of the spinal cord [21]. Some studies have been published concerning the treatment of SCI injuries in human patients [22-24].

In 2016, the team led by Dai reported promising results after the transplant of collagen-based scaffolds in human patients with complete SCI at the cervical or thoracic level. According to their study, the resection of the scar or the scaffold transplant did not have any easily adverse effects. They reported improved penis reflex two months after the surgery; while in two cases, there was recovery of somatosensory evoked potentials six months after the operation. Additionally, skin sweating was partially recovered below the level of the injury in three cases [23].

In 2018, the same team performed transplants of collagen-based scaffolds with human umbilical mesenchymal stem cells in two cases of SCI injuries in humans. The first case, where the injury was at the T11 level, showed recovery of sensory function at two months, which was further improved at six months. They also reported sense function of the bowel and the bladder at nine months. The muscle function was progressively regained below the T11 level after the injury, and at 12 months, the patient could walk with a brace. The Cervical SCI patient began to recover sensory function at two months and increased up to S5 at nine months. At 12 months post-surgery, the patient regained accurate bowel and bladder sensory function. The muscle control also improved, and at six months, the patient could even raise his lower legs against gravity. In both cases, the ASIA score improved from A to C 12 months post-surgery [22].

The importance of animal models in SCI studies

Al-Hoseini et al., noticing the importance of using animal models in SCI studies, conducted a systematic review on “Animal Models of Spinal Cord Injury”. The researchers categorized 2,209 injuries according to level, outcome, animal species and purpose of the study. Most of the reviewed studies examined drug effectiveness, while others observed pathophysiologic changes. Eighty-one per cent of SCI sites involved the thoracic region, whilst contusion, transection and compression were the most common injury types induced. The majority (72.4%) of SCI assessments were conducted on rats, as the rodents' biological and behavioural outcomes and biomechanics and neuropathology highly resemble humans. According to the study, rodents, such as mice or rats, are optimal for preliminary SCI studies because of the low reproductory cost and resemblance to human beings in terms of pathology and genomes [25]. Cats are another popular choice in SCI studies, mainly because they are larger than rodents, allowing more effortless surgical manoeuvres. Another important preclinical model is the pig, which combines an intermediate size and a more remarkable resemblance to human physiology. Fish, lamprey and other vertebrates have also been deployed in novel SCI studies, owing to their unique regeneration capability. The study points out that the optimal choice for SCI studies would be the non-human primates and larger animals that represent human SCIs a lot better than other organisms [25]. Notwithstanding the above, these primates are not ultimately deployed in such studies because of costly care and regulatory and ethical considerations. As an alternative, canines can be studied in laboratory conditions after naturally occurring SCI (e.g., due to accidents), causing less moral concern.

As shown in Table 1, four studies [14-16,26] carried out between 2014 and 2017 have applied collagen-based scaffolds for SCI treatment in mouse, rat and canine models. In these cases, the stump connection, the transplanted cell differentiation, the elimination of glial scar and the increased neuronal growth were noted. Additionally, the decreased collagen deposition, the behavioural recovery, the improved electrophysiology and the enhanced axonal regeneration were evident.

**Table 1:** Review of SCI studies deploying collagen-based scaffolds in animal models

Material	Animal model	Spinal cord injury type	Outcome	Functional recovery		Reference
				Motor	Sensory	
Collagen	Mouse	Transection	Connection of stumps in the transected spinal cord	N	NM	Sugai et al. (2015) [15]
	Rat	Hemisection	Decrease of glial scaring and collagen deposition, increase of neurons	Y	NM	Breen et al. (2016) [14]
	Canine		Recovery of behavioral and electrophysiology: preventing formation of glial scars; enhancing axon regeneration	Y	Y	Han et al. (2014,2017)

## Conclusions

This review has aimed to compile the latest bibliographical data available concerning the application of collagen scaffolds to treat SCI in animal models. SCI is one of the most critical cases a patient and a surgeon may encounter, bearing significant economic and psychological implications. A few relevant studies have recently been carried out using carefully selected animals that resemble human pathophysiology. Collagen-based scaffold application is one of the most promising methods of SCI treatment.
